# Association between family nurturing environment and screen exposure among preschool children aged 3–6 years in Shanghai: A cross-sectional study

**DOI:** 10.1371/journal.pone.0337043

**Published:** 2026-03-10

**Authors:** Min Chen, Yun Li, Shurong Kang, Chunhua Jiang, Yinan Liu, Jian Li

**Affiliations:** 1 Department of Children Health Care, Minhang District Maternal and Child Healthcare Hospital, Shanghai, China; 2 Clinical Research Center, Ruijin Hospital, Shanghai Jiao Tong University School of Medicine, Shanghai, China; No institution, UNITED KINGDOM OF GREAT BRITAIN AND NORTHERN IRELAND

## Abstract

**Background:**

Excessive or early screen exposure has been deemed associated with both immediate and long-term health impairment among preschool children. This study aimed to investigate the association between family nurturing environment and screen exposure among preschool children aged 3–6 years.

**Materials and methods:**

A multi-stage stratified cluster sampling method was utilized to sample the kindergarten children aged 3-6 years from a district in Shanghai, China. From April to May 2023, parents completed an online questionnaire. Children's screen exposure was defined as more than 1 hour (hr) per day of screen-based devices use, and the daily screen time was categorized into three groups: non-exposure (<1hr/ day), low exposure (1-4 hrs/ day), and high exposure (>4 hrs/ day). Univariate and multivariate cumulative logit regression models were adopted to identify the determinants of screen exposure.

**Results:**

A total of 1917 preschool children were included. Of these, 1604 (83.7%) were exposed to screens for more than 1 hr per day. High, low, and non-exposure groups comprised 313 (16.3%), 1291 (67.4%), and 313 (16.3%) children, respectively. The multivariate cumulative logit model showed that parental education, monthly household income, presence of screen-based devices in the bedroom, and parental screen use in front of children were positive determinants of more severe screen exposure for preschoolers. Conversely, older age, absence of siblings, co-viewing with the child, and parental restriction of screen time were negative determinants of more severe screen exposure.

**Conclusions:**

Our findings suggest that interventions targeting parental behavior and cognitive practices may be more effective in promoting healthy screen exposure habits in preschool children.

## Introduction

The family nurturing environment refers to the nurturing setting provided by the family for children during their early life stage. As a fundamental and important context for early child development, it generally comprises both subjective dimensions, including parental attitudes, concepts toward children, and nurturing behaviors, and objective dimensions, including household income, parental educational background, and family structure.

Over the past decades, there has been a noteworthy increase in the diversity and use of screen-based devices, which have infiltrated into every aspect of many children’s lives [1]. Screen-based devices accessible to young children now include not only traditional televisions (TV) and computers but also new media such as smartphones, tablets and game consoles. Screen exposure in early childhood has become a growing public health concern worldwide [2]. The World Health Organization (WHO) recommends that screen exposure for children younger than 18 months should be avoided, and that screen time for children aged 2–5 years be limited to no more than one hour (hr) per day [3]. Nevertheless, daily screen time among children is increasing, while the age of first exposure is decreasing [4]. A study by Zimmerman et al. [5] revealed that 90% of children in the U.S. had already been exposed to TV screens by the age of 2. Excessive screen exposure in children is associated with numerous negative consequences, including reduced physical activity, obesity, sleep disturbances, attention deficits, aggressive behavior, language and cognitive delay, and poor academic performance [6–9]. Furthermore, it has been well documented that negative health outcomes in later years may be predicted by adverse screen exposure habits shaped early in life [10]. Therefore, promoting appropriate screen exposure in preschoolers may be critically important for their lifelong health and well-being.

A prerequisite for developing scientific interventions to reduce excessive screen exposure in preschoolers is understanding the determinants that influence this behavior. However, relatively little is known about the relationships between factors within the family nurturing environment and screen exposure of preschool children (i.e., those 3–6 years old), especially in non-Western contexts like China [11]. In China, and particularly in highly developed megacities such as Shanghai, unique cultural factors—such as intensive parenting styles focused on academic achievement, the prevalent use of technology for early education, and the common involvement of grandparents in childcare—may shape distinct patterns of screen media use among preschool children. These cultural specificities highlight the significance of generating localized evidence to inform effective guidelines and interventions. To address this knowledge gap, the current study aims to analyze the impacts of relevant factors in the family nurturing environment on screen exposure among kindergarten children in an urban district of Shanghai, China.

## Materials and methods

### Study design and participants

This cross-sectional observational study was carried out from April 4, 2023 to May 27, 2023 using a multi-stage stratified cluster sampling method. In the first stage, 7 sub-districts were randomly selected from all 14 sub-districts in the target district. In the second stage, three kindergartens were randomly selected from each chosen sub-district. In the last stage, one junior, one middle, and one senior class were randomly selected from each kindergarten. All children in these classes were considered potentially eligible participants. The inclusion criteria were: being a full-time student in one of the selected kindergartens, and aged between 3 and 6 years. Children who had a history of prematurity, low birth weight, language development delay, or major congenital diseases were ruled out. The study was conducted in compliance with the Declaration of Helsinki (as revised in 2013) and was approved by the Ethics Committee of Minhang District Maternal and Child Healthcare Hospital under approval number (2021KS-02, dated 13 May, 2021). Written informed consent was obtained from all guardians of children. The study followed the guidelines of Strengthening the Reporting of Observational Studies in Epidemiology (STROBE).

### Data collection

Teachers of the selected classes sent an invitation message and a QR code linked to the online questionnaire to parents via WeChat groups used for class communication. The structured questionnaire, which consisted entirely of close-ended questions, was designed by the research team and pilot-tested prior to formal investigation. It was to be completed within approximately 10 minutes (min) by the caregiver most familiar with the child and submitted online. Demographic and socioeconomic information collected included the child’s gender, birth date, presence of siblings, family structure (coded as 1 = two-parent family; 2 = parent-grandparent; 3 = single-parent family), household registration (Shanghai or other province/municipality), parental education (categorized into 4 levels from high school to master/doctor), and monthly household income (grouped into 4 categories from ‘<10000 RMB’ to ‘>50000 RMB’).

Caregivers provided self-reported estimates of the child’s screen exposure time on weekdays and weekends, respectively by answering the question: “How many hrs per typical day (separated into weekdays and weekends) did your child spend on screen-based devices (including TV, video games, smartphones, tablets, etc.) over the past 7 days?” The following formula was adopted to calculate the mean amount of daily screen time: (weekend screen time×2 + weekday screen time×5)/7. Additional information collected included the age at first screen exposure, the type of screen-based device most frequently used, and whether such devices were present in the child’s bedroom. Parents were also questioned regarding their own behaviors and perceptions related to screen exposure, such as whether they used recreational screen devices in front of the child, engaged in co-viewing, restricted the child’s screen time, or held favorable attitudes toward the child’s use of screen devices.

### Statistical analysis

According to our pilot survey conducted in a kindergarten with a sample of 150 caregivers of preschool children (50 from each of the junior class, middle, and senior classes), the mean daily screen time among preschoolers aged 3–6 years was 2.2 hrs with a standard deviation (SD) of 1.6 hrs. Based on these data, a minimum sample size of 903 was calculated, assuming a precision of 5%, a confidence level of 95%, and an anticipated dropout rate of 10%. In view of the multi-stage cluster sampling technique reducing efficiency and precision, a design effect (DEFF) of 2.0 was applied. Thus, a final sample size of 1806 was deemed sufficient for this study.

Continuous variables are expressed as the mean ±SD and were compared by the independent *t* test or the paired *t* test, as appropriate. Categorical variables are presented as frequencies with proportions, and group differences were assessed using the Pearson chi-square test. Missing values for variables were imputed via the expectation-maximization algorithm. The specific variables with missing data and their respective percentages were as follows: mother’s education (1.04%), father’s education (1.25%), and monthly household income (2.24%). No other variables contained missing data, as questionnaires were confirmed for completeness during data collection. In this study, daily screen time ≥1 hr was defined as screen exposure [12]. Children were categorized into three groups based on daily screen time: non-exposure (<1 hr/day), low exposure (1–4 hrs/day), and high exposure (>4 hrs/day) [9].

To explore determinants associated with the severity of screen exposure, we employed an ordinal outcome variable (non, low, high screen exposure) and constructed cumulative logit models. The univariate and multivariate cumulative logit models were fitted sequentially, with non-screen exposure as the reference. Potential factors were examined using univariate ordinal logistic regression initially. Variables that were not significant in the univariate analysis were excluded from the multivariate model. Interaction effects were not analyzed to maintain model parsimony and preserve statistical power for testing primary hypotheses. Adjusted odds ratios (OR) and 95% confidence intervals (CI) were calculated. The statistical analyses were conducted using SPSS version 25.0 (IBM Corp., Armonk, NY, USA). All tests were performed two-sided at the 5% significance level.

## Results

### Participant characteristics

Of the 2031 caregivers invited to participate, 76 (3.74%) declined and 38 (1.87%) submitted incomplete questionnaires lacking the critical information of the child’s date of birth. Finally, data from 1917 caregivers were included in the analysis. Demographic and socioeconomic characteristics are presented in Table 1. Just over half (52.5%) of the children were male, with a mean (SD) age of 61.92 (10.78) months. A total of 1067 (55.7%) children were the only child in their family, and 76.6% had household registration in Shanghai. Regarding family structure, 58.8% of children lived in parent-grandparent households, and 40.4% in two-parent households. Furthermore, 69.9% of mothers and 71.3% of fathers held a bachelor’s degree or higher. Approximately 62.9% of the families reported a monthly household income >20000 RMB. In terms of device usage, the majority of preschool children (66.4%) used tablets/iPads, while 30.4% used smartphones. As also shown in Table 1, 23.8% of children had screen devices in their bedroom. Up to 89.4% of parents reported using recreational screen devices in front of their children, and 71.8% of parents engaged in co-viewing with their children. Although 95.5% of parents indicated that they restricted their child’s screen time, 38.1% expressed a favorable attitude toward children’s use of screen devices.

**Table 1:** Participant characteristics in different exposure groups.

Variables	n(%)	Screen exposure			*P* value
		Non	Low	High	
Child’s gender					0.756*
Male	1006(52.5)	167(53.4)	670(51.9)	169(54.0)	
Female	911(47.5)	146(46.6)	621(48.1)	144(46.0)	
Child’s age(months),mean±SD	61.92 ± 10.78	60.77 ± 10.88	61.90 ± 10.84	63.17 ± 10.30	0.020^#^
Child’s age(years)					0.027*
3	211(11.0)	45(14.4)	148(11.5)	18(5.8)	
4	597(31.1)	102(32.6)	396(30.7)	99(31.6)	
5	631(32.9)	95(30.4)	426(33.0)	110(35.1)	
6	478(24.9)	71(22.7)	321(24.9)	86(27.5)	
Presence of siblings					0.001*
No	1067(55.7)	194(62.0)	726(56.2)	147(47.0)	
Yes	850(44.3)	119(38.0)	565(43.8)	166(53.0)	
Household registration					0.283*
Shanghai	1469(76.6)	250(79.9)	985(76.3)	234(74.8)	
Other province	448(23.4)	63(20.1)	306(23.7)	79(25.2)	
Family structure					0.542*
Two-parent	775(40.4)	135(43.1)	512(39.7)	128(40.9)	
Parent-grandparent	1128(58.8)	176(56.2)	771(59.7)	181(57.8)	
Single-parent	14(0.7)	2(0.6)	8(0.6)	4(1.3)	
Mother’s education					<0.001*
Senior high school and less	184(9.6)	10(3.2)	113(8.8)	61(19.5)	
Junior college	394(20.6)	51(16.3)	267(20.7)	76(24.3)	
Bachelor	971(50.7)	169(54.0)	664(51.4)	138(44.1)	
Master and higher	368(19.2)	83(26.5)	247(19.1)	38(12.1)	
Father’s education					<0.001*
Senior high school and less	171(8.9)	4(1.3)	110(8.5)	57(18.2)	
Junior college	379(19.8)	41(13.1)	251(19.4)	87(27.8)	
Bachelor	938(48.9)	174(55.6)	639(49.5)	125(39.9)	
Master and higher	429(22.4)	94(30.0)	291(22.5)	44(14.1)	
Monthly household income					<0.001*
Less than 10000 RMB	173(9.0)	12(3.8)	104(8.1)	57(18.2)	
10000RMB-20000 RMB	537(28.0)	84(26.8)	348(27.0)	105(33.5)	
20001RMB-50000 RMB	831(43.3)	147(47.0)	587(45.5)	97(31.0)	
More than 50000 RMB	376(19.6)	70(22.4)	252(19.5)	54(17.3)	
Age at first screen exposure					0.538*
≤12 months	615(32.1)	94(30.0)	414(32.1)	107(34.2)	
>12 months	1302(67.9)	219(70.0)	877(67.9)	206(65.8)	
Screen-based devices in the bedroom					<0.001*
Yes	457(23.8)	42(13.4)	311(24.1)	104(33.2)	
No	1460(76.2)	271(86.6)	980(75.9)	209(66.8)	
Use of recreational screen devices in front of the child					0.006*
Yes	1714(89.4)	264(84.3)	1165(90.2)	285(91.1)	
No	203(10.6)	49(15.7)	126(9.8)	28(8.9)	
Co-viewing with the child					<0.001*
Yes	1377(71.8)	259(82.7)	929(72.0)	189(60.4)	
No	540(28.2)	54(17.3)	362(28.0)	124(39.6)	
Restricting the child’s screen time					<0.001*
Yes	1831(95.5)	307(98.1)	1239(96.0)	285(91.1)	
No	86(4.5)	6(1.9)	52(4.0)	28(8.9)	
Having a favorable attitude toward screen device use					0.003*
Yes	730(38.1)	93(29.7)	519(40.2)	118(37.7)	
No	1187(61.9)	220(70.3)	772(59.8)	195(62.3)	

*: Pearson Chi-square test ^#^:independent *t*-test

### Children’s screen exposure time

**Fig 1:**
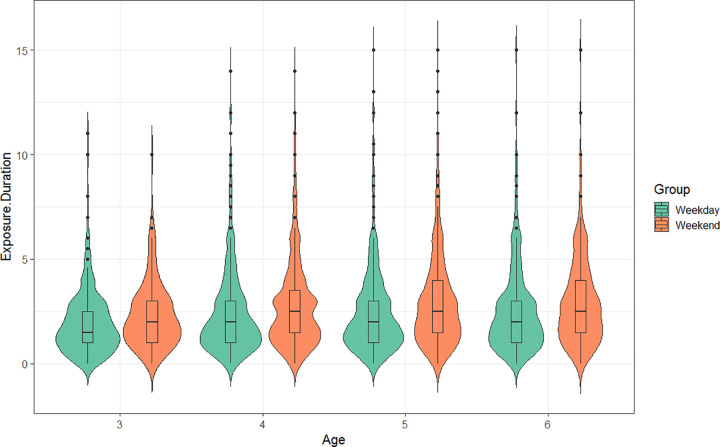
Screen time duration on weekdays and weekends, stratified by age. Box plots shows the distribution of daily screen time (in hrs) for children aged 3–6 years, comparing weekdays (teal) and weekends (orange). Within each box plot, the central line represents the median, the box spans the interquartile range (25th to 75th percentile), and the whiskers extend to the minimum and maximum values within 1.5 times the interquartile range. Individual data points are outliers. The y-axis shows the exposure duration (hrs) and ranges from 0 to 15. The x-axis shows the age (years). For all age groups, screen time was significantly longer on weekends compared to weekdays (P < 0.001).

In the current study, a total of 1604 children (83.7%) were exposed to screens for more than 1hr per day. Among them, 1291 (67.4%) had daily screen time of 1–4 hrs, and 313 (16.3%) exceeded 4 hrs; the remaining 313 (16.3%) had no screen exposure. The average daily screen time among preschoolers was 2.54 ± 1.99 hrs. Screen time was significantly higher on weekends (2.84 ± 2.13 hrs/day) compared to weekdays (2.42 ± 2.05 hrs/day) (*P* < 0.001). Screen time stratified by age on weekdays and weekends is detailed in Table 2. The distribution of screen time on weekdays and weekends is presented in Fig 1.

**Table 2:** Screen exposure time of preschool children on weekday and weekend.

Age (years)	Screen time on weekday	Screen time on weekend	*P* value*
3 ys	2.00 ± 1.64	2.30 ± 1.57	0.042
4 ys	2.43 ± 2.06	2.79 ± 2.12	0.001
5 ys	2.46 ± 2.05	2.95 ± 2.22	<0.0001
6 ys	2.54 ± 2.12	2.97 ± 2.15	0.001

*: Paired *t*-test

### Determinants of screen exposure for children

A total of 14 variables were initially included in univariate ordinal logistic regression analysis; results are exhibited in Table 3. Ten variables were found to be significantly associated with screen exposure, including child’s age, presence of siblings, mother’s education, father’s education, monthly household income, presence of screen-based devices in the bedroom, parental use of recreational screen devices in front of the child, co-viewing, restricting screen time, and having a favorable attitude toward screen device use（Table 3）.

**Table 3:** Results of univariate ordinal logistic model using severity of screen exposure as the response.

Variables	Level	β	Crude OR(95%CI)	*P* value
Child’s gender	Female	Ref		
	Male	0.015	1.015(0.842-1.225)	0.873
Child’s age	3 ys	−0.572	0.564(0.403-0.791)	0.001
	4 ys	−0.134	0.875(0.679-1.125)	0.296
	5 ys	−0.028	0.972(0.758-1.247)	0.824
	6 ys	Ref		
Presence of siblings	No	−0.366	0.694(0.573-0.839)	<0.001
	Yes	Ref		
Household registration	Shanghai	−0.170	0.844(0.676-1.052)	0.132
	Other province	Ref		
Family structure	Two-parent	−0.577	0.562(0.190-1.662)	0.297
	Parent-grandparent	−0.528	0.590(0.2500-1.742)	0.339
	Single-parent	Ref		
Mother’s education	Senior high school and less	1.472	4.358(3.010-6.309)	<0.001
	Junior college	0.700	2.014(1.490-2.724)	<0.001
	Bachelor	0.338	1.402(1.090-1.806)	0.009
	Master and higher	Ref		
Father’s education	Senior high school and less	1.557	4.745(3.274-6.869)	<0.001
	Junior college	0.910	2.484(1.848-3.340)	<0.001
	Bachelor	0.243	1.275(1.001-1.623)	0.049
	Master and higher	Ref		
Monthly household income	Less than 10000 RMB	1.115	3.050(2.101-4.428)	<0.001
	10000RMB-20000 RMB	0.310	1.363(1.033-1.802)	0.029
	25001RMB-50000 RMB	−0.061	0.941(0.728-1.215)	0.640
	More than 50000 RMB	Ref		
Age at first screen exposure	≤12 months	0.114	1.121(0.917-1.370)	0.266
	>12 months	Ref		
Screen-based devices in the bedroom	Yes	0.654	1.923(1.540-2.401)	<0.001
	No	Ref		
Use of recreational screen devices in front of the child	Yes	0.433	1.542(1.139-2.088)	0.005
	No	Ref		
Co-viewing with the child	Yes	−0.668	0.513(0.415-0.633)	<0.001
	No	0		
Restricting the child’s screen time	Yes	−0.967	0.380(0.245-0.589)	<0.001
	No	Ref		
Having a favorable attitude toward screen device use	Yes	0.201	1.223(1.008-1.483)	0.041
	No	Ref		

Variables significant in the univariate analysis were incorporated into a multivariable cumulative logit model. Nine variables remained statistically significant predictors of screen exposure in the final model; only “favorable attitude toward screen device use” was not retained (Table 4). Results revealed that children whose mothers had a high school education or less were at 1.797 times higher risk of more severe screen exposure (95% CI: 1.135–2.846) compared to those whose mothers held a master’s degree or higher. Similarly, children whose fathers had a high school education or a junior college degree had 2.782 (95% CI: 1.770–4.367) and 1.779 (95% CI: 1.247–2.535) times higher risk, respectively, when compared with children whose fathers had a master's degree or higher. Children from households with a monthly income <10000 RMB were at higher risk than those with >50000 RMB (OR = 2.257; 95% CI: 1.517–3.357). Children with screen-based devices in their bedroom had a 1.781-fold greater risk of severe screen exposure (95%CI: 1.415–2.241) compared to those without screen devices in the bedroom. Parental use of recreational screen devices in front of the child was also associated with a more severe risk (OR = 1.499; 95% CI: 1.097–2.050). In contrast, 3-year-old children and only children had lower risks of having more severe screen exposure, with ORs of 0.675 (95% CI: 0.477–0.955) and 0.705 (95% CI: 0.579–0.859), respectively, compared to 6-year-olds and children with siblings. Additionally, parental co-viewing (OR = 0.618; 95% CI: 0.497–0.769) and restricting screen time (OR = 0.516; 95% CI: 0.326–0.816) were independently protective factors. The Hosmer-Lemeshow test indicated that the model had superior goodness-of-fit (χ^2^ = 2122.55, *P* = 0.879), and the Pseudo-R^2^ was 0.135.

**Table 4:** Results of multivariate ordinal logistic model using severity of screen exposure as the response.

Variables	Level	β	Adjusted OR(95%CI)	*P* value
Child’s age	3 ys	−0.393	0.675(0.477-0.955)	0.026
	4 ys	−0.035	0.966(0.746-1.251)	0.793
	5 ys	0.059	1.061(0.823-1.366)	0.651
	6 ys	Ref		
Presence of siblings	No	−0.349	0.705(0.579-0.859)	0.001
	Yes	Ref		
Mother’s education	Senior high school and less	0.586	1.797(1.135-2.846)	0.012
	Junior college	0.263	1.301(0.907-1.866)	0.153
	Bachelor	0.170	1.185(0.882-1.590)	0.259
	Master and higher	Ref		
Father’s education	Senior high school and less	1.023	2.782(1.770-4.367)	<0.001
	Junior college	0.576	1.779(1.247-2.535)	0.001
	Bachelor	0.095	1.100(0.830-1.456)	0.508
	Master and higher	Ref		
Monthly household income	Less than 10000 RMB	0.814	2.257(1.517-3.357)	<0.001
	10000RMB-20000 RMB	0.219	1.245(0.932-1.664)	0.138
	25001RMB-50000 RMB	0.013	1.013(0.779-1.318)	0.922
	More than 50000 RMB	Ref		
Screen-based devices in the bedroom	Yes	0.577	1.781(1.415-2.241)	<0.001
	No	Ref		
Use of recreational screen devices in front of the child	Yes	0.405	1.499(1.097-2.050)	0.011
	No	Ref		
Co-viewing with the child	Yes	−0.481	0.618(0.497-0.769)	<0.001
	No	Ref		
Restricting the child’s screen time	Yes	−0.662	0.516(0.326-0.816)	0.005
	No	Ref		

## Discussion

With the progress of multimedia technologies, Screen-based devices such as TVs, smartphones and tablets have become ubiquitous in households worldwide, and children are spending more time on these devices than ever before. Although global disparities in screens and electronic media access persist—for example, less than half of the population in 71 out of 195 countries globally can access to the internet—it is undeniable that global Internet usage is on the rise, especially among children [13]. More and more preschool children get acquainted with smartphones and tablets before mastering basic life skills, leading to a tendency of earlier initiation and excessive screen exposure time [14,15]. Tremblay et al. [16] conducted a survey across 38 countries on children's screen exposure, and found that 60% to 93% of children within 35 of these countries were exposed to more than 2 hrs of screen time per day. Similarly, an Australian survey reported that children aged 3–5 years spent an average of 2 hrs and 13 min on screen daily [17]. Presently, there is very limited existing literature on screen exposure among children in China. A recent study conducted in Beijing reported that the daily screen time of children aged 1.5–3 years was 1.2 hrs [18]. Similar to the findings from developed countries, the present study demonstrates that preschoolers aged 3–6 in Shanghai average 2.54 hrs of daily screen exposure. It is well established that excessive screen time or too early exposure to electronic media—particularly when displacing emotionally rich interactions with parents—can adversely affect children’s physical and mental development. Preschoolers, especially those aged 3–6 years, are in a critical developmental period during which their health is vulnerable to external influences. Therefore, the relationship between screen time and preschool children's health warrants serious attention. The guidelines issued by the American Academy of Pediatrics (AAP) define screen time exceeding 1 hr per day as excessive for children aged 2–5 years and recommend that using screen devices should occur under parental supervision [9]. In China, the 2018 Physical Activity Guidelines for Chinese Children and Adolescents, developed by a national working group, recommend that screen time for children should be limited to less than 2 hrs per day. This represents the first official guideline on children’s screen exposure in China. Our study found that screen time increased with age and was significantly higher on weekends than on weekdays across all age groups. Earlier and excessive exposure in children under 6 years of age to screens may disrupt brain development and negatively affect language use and acquisition, attention, cognitive development, executive function, and overall health. Parents should enhance the quantity and quality of parent-child interaction and reduce screen exposure among preschool children.

The present study demonstrates that the risk of screen exposure among preschoolers is independently determined by multiple factors at the levels of both the individual and the family nurturing environment. As described previously [19], younger preschool children were at lower risk of more severe screen exposure, with risk increasing with age. It has been reported that parents with higher education attainment use screen-based devices less frequently in front of children, and place greater emphasis on limiting children’s screen time compared to those with low education levels [20]. In this study, children whose parents had only a high school education showed the highest risk of severe screen exposure. Parental education level was inversely correlated with the risk of screen exposure in preschoolers, aligning with previous studies [21,22]. Similarly, the present study revealed that monthly household income was an independently negative predictor of severe screen exposure, with children from lower-income households being more likely to experience more severe screen exposure, consistent with previous reports [19]. Conflicting evidence exists with regard to the association between screen time and the presence of siblings [22]. Hinkley et al. [23] found no relationship between the presence of siblings and screen time in preschoolers. On the other hand, Oflu et al. [9] identified having siblings as a positive determinant of screen time. Aligning with the latter, we found that being an only child was associated with a lower risk of more severe screen exposure compared to having siblings. This may reflect multifactorial dynamics: the presence of siblings perhaps can increase parental caregiving burden, reduce the quality time of parent-child interaction, and thereby lead to excessive screen exposure for children. Another possible explanation is the role-modeling effect among siblings, wherein older siblings may introduce screen habits to younger ones through imitation—an influence that might outweigh parental monitoring efforts. Additionally, shared screen use among siblings (e.g., watching videos or playing games together) may artificially inflate individual screen time, even under parental supervision.

Parents’ behavioral and cognitive factors within the home environment also exert independent and significant impacts on preschoolers’ screen exposure. Existing studies have demonstrated that having screen-based devices such as TV in the child’s bedroom is an independent predictor of screen exposure [19]. Our study confirmed that preschoolers with screen devices in the bedroom were at higher risk of severe screen exposure. Using electronic media before bedtime exposes children to screen light that can stimulate the eyes, suppress melatonin secretion, and thus interfere with sleep. Therefore, AAP guidelines clearly recommend against placing a TV in children's bedrooms. Previous studies have also shown a positive correlation between the amount of parental screen time and that of their children’s screen time [19,24]. Our study further demonstrated that the risk of more severe screen exposure in preschoolers significantly increased when their parents used screen devices in front of them. Such parental behavior may impede positive parent-child communication and, given children’s tendency to imitate, may encourage premature and excessive screen use. Preschoolers who view screens alone were inclined to be more emotionally fluctuous. AAP therefore suggests that parents co-view with children and help them interpret screen content. Previous studies have identified parental co-viewing with children as a protective factor for preschoolers’ screen exposure that can reduce screen time [9], a finding supported by our results. Children whose parents co-viewed screen devices with them were less likely to experience more severe screen exposure. A research conducted in the Philippines [25] similarly identified the absence of co-viewing as a significant predictors of increased screen time among preschoolers. Specifically, children who watched screens alone had 8.56 times greater odds of excessive screen use compared to those who co-viewed with a parent. When comparing our patterns with findings from foreign contexts, it is important to consider unique socio-cultural norms in Shanghai. For instance, the high prevalence of three-generational households may shape distinct screen exposure patterns, as grandparents might often utilize screens for distraction or educational purposes, potentially leading to more permissive screen use norms compared to nuclear family structures common elsewhere. Additionally, our study demonstrated that parental restriction of preschoolers’ screen time was an independently protective determinant, associated with a reduced risk of more severe screen exposure. In line with our results, some research indicated that parents’ restriction regarding the use of screens was linked to children spending less time on screens [26]. These consistent findings across diverse cultural contexts strengthen the evidence base for developing targeted interventions aimed at modifying parental screen-related behaviors—such as promoting co-viewing and setting screen time limits—as potential strategies for future interventions.

To the best of our knowledge, the strengths of this study encompass its large sample size and its status as one of the first internet-based investigations into determinants of screen exposure in preschool children in China. Furthermore, the 3-tier classification of screen exposure enabled a more nuanced analysis beyond a simple binary outcome (exposure/non-exposure), revealing potential gradient effects, and offering deeper insights into the association between different levels of screen exposure and various family nurturing factors. Nevertheless, several limitations in this study should be acknowledged. First, the cross-sectional design inherently limits the ability to infer causality or directionality. Without longitudinal data, the identified associations may reflect correlation, reverse causality, or unmeasured confounding rather than true causal relationships due to the absence of temporal sequencing and potential biases like simultaneity. Future intervention studies, such as randomized controlled trials testing strategies to optimize parental screen use rules, promote screen-free parenting practices, or limit device accessibility, could help establish causality by comparing changes in screen time between intervention and control groups. Such designs would help clarify whether observed associations represent true causal effects or are influenced by unmeasured familial or socioeconomic factors. Second, screen exposure time of preschoolers was reported by parents, which could be subject to recall bias and social desirability bias. Parents may under-report screen time due to awareness of guidelines or perceived stigma associated with high use, leading to systematic underestimation of actual screen exposure levels. Future research should incorporate objective measures of screen time to mitigate biases inherent in caregiver self-reports. For instance, screen time tracking apps, device usage logs, or wearable sensors could provide more precise and real-time data. Combining objective measures with parental reports would allow cross-validation and improve reliability, thereby enhancing the validity of findings and supporting the development of effective interventions. Third, the current study was conducted in a single district in Shanghai, which limited the extrapolation of findings to a certain extent. Future studies should adopt multi-center designs across various Chinese cities and comparative international collaborations to better understand both universal and culturally specific determinants of screen time in preschool children.

## Conclusions

This study identified a relatively high prevalence of screen exposure among preschool children aged 3–6 years. The family nurturing environment was found to be particularly relevant to excessive screen exposure of children. Nonetheless, due to the cross-sectional nature of our study, the observed associations between family nurturing environment and children’s screen exposure may reflect complex, bidirectional influences. It is plausible that while family environment may influence screen time, increased screen exposure may also reciprocally reduce opportunities for parent-child interaction and thus shape the family nurturing environment. In the absence of rapid improvements in objective family conditions such as socioeconomic status, intervention strategies focused on modifying parental behaviors and perceptions may represent a more feasible and effective approach to fostering healthy screen exposure habits in preschool children.

## Supporting information

S1 File Study data. (XLSX)
